# Use of physician billing claims to identify infections in children

**DOI:** 10.1371/journal.pone.0207468

**Published:** 2018-11-12

**Authors:** Jeremiah Hwee, Lillian Sung, Jeffrey C. Kwong, Rinku Sutradhar, Karen Tu, Jason D. Pole

**Affiliations:** 1 Dalla Lana School of Public Health, University of Toronto, Toronto, Ontario, Canada; 2 Institute for Clinical Evaluative Sciences, Toronto, Ontario, Canada; 3 Department of Pediatrics, Division of Haematology/Oncology, The Hospital for Sick Children, Toronto, Ontario, Canada; 4 Program in Child Health Evaluative Sciences, The Hospital for Sick Children, Peter Gilgan Centre for Research and Learning, Toronto, Ontario, Canada; 5 Institute of Health Policy, Management and Evaluation, University of Toronto, Toronto, Ontario, Canada; 6 Department of Family and Community Medicine, University of Toronto, Toronto, Ontario, Canada; 7 Public Health Ontario, Toronto, Ontario, Canada; 8 Toronto Western Family Health Team, University Health Network, Toronto, Ontario, Canada; 9 Pediatric Oncology Group of Ontario, Toronto, Ontario, Canada; Rabin Medical Center, Beilinson Hospital, ISRAEL

## Abstract

While medical records have detailed information, they are limited in reach to the availability and accessibility of those records. On the other hand, administrative data while limited in scope, have a much further reach in coverage of an entire population. However, few studies have validated the use of administrative data for identifying infections in pediatric populations. Pediatric patients from Ontario, Canada aged <18 years were randomly sampled from the Electronic Medical Record Administrative data Linked Database (EMRALD). Using physician diagnoses from the electronic medical record (EMR) as the reference standard, we determined the criterion validity of physician billing claims in administrative data for identifying infectious disease syndromes from 2012 to 2014. Diagnosis codes were assessed by infection category (respiratory, skin and soft tissue, gastrointestinal, urinary tract and otitis externa) and for all infections combined. Sensitivity analyses assessed the performance if patients had more than one reason to visit the physician. We analysed 2,139 patients and found 33.3% of all visits were for an infection, and respiratory infections accounted for 67.6% of the infections. When we combined all infection categories, sensitivity was 0.74 (95% CI 0.70–0.77), specificity was 0.95 (95% CI 0.93–0.96), positive predictive value (PPV) was 0.87 (95% CI 0.84–0.90), and negative predictive value (NPV) was 0.88 (95% CI 0.86–0.89). For respiratory infections, sensitivity was 0.77 (95% CI 0.73–0.81), specificity was 0.96 (95% CI 0.95–0.97), PPV was 0.85 (95% CI 0.81–0.88), and NPV was 0.94 (95% CI 0.92–0.95). Similar performance was observed for skin and soft tissue, gastrointestinal, urinary tract, and otitis externa infections, but with lower sensitivity. Performance measures were highest when the patient visited the physician with only one health complaint. We found when using linked EMR data as the reference standard, administrative billing codes are reasonably accurate in identifying infections in a pediatric population.

## Introduction

Healthcare administrative data provide a rich source of population-based information. However, since the data are passively collected for administrative purposes rather than for research, validation studies are necessary to determine the accuracy of these data for identifying diseases. Infections are the most frequent reason reported for seeking healthcare in children and adolescents aged <18 years, accounting for the majority of emergency department and physician office visits.[1–4] Using administrative data to study infections would be advantageous, allowing large populations of children to be studied efficiently. However, few studies have validated the use of administrative data for identifying infections in pediatric populations.

Ontario is Canada’s most populous province, with a population of 13.9 million as of 2016, including 2.6 million residents aged <18 years.[5] Because of the single-payer healthcare system, almost all encounters with the system are captured in province-wide administrative databases. The data are accurate for identifying other pediatric diseases such as diabetes and asthma, as well as receipt of immunizations.[6–8] Our objective was to assess the criterion validity of administrative data for identifying infections compared to electronic medical records (EMR) data as the reference standard.

## Methods

The study was approved by University of Toronto’s Health Sciences Research Ethics Board and Sunnybrook Health Sciences Centre’s Research Ethics Board. The Institute of Clinical Evaluative Sciences (ICES) is named as a prescribed entity under provincial privacy legislation. Under this designation, ICES can receive and use health information without consent for the purposes of health-related research and health system analysis and evaluation conducted by ICES, independently or on behalf of policy-makers or other stakeholders.

### Study design, population, and setting

We conducted a validation study of infectious disease billing codes submitted by physicians compared to the reference standard of infections documented in a primary care EMR. We sampled a random cohort of Ontario residents aged <18 years who were under the care of family physicians who share their practice’s EMR data with the Electronic Medical Record Administrative data Linked Database (EMRALD). Patient visits between April 1, 2012 and March 31, 2014 were randomly chosen for extraction and verification. The globally unique identifier approach was used to generate a random sample using Microsoft SQL Server Management Studio 2012 (Microsoft Corporation). We limited patients to only one visit to minimize the impact of multiple visits for the same illness.

We used an intermediate-prevalence estimate to determine the sample size for the infectious syndromes with the goal to validate any infection. The estimated annual prevalence of otitis media infections in a pediatric population was 11.5% in Ontario.[9] Using the binomial distribution, we needed 2,044 patients, with 235 patients with otitis media infections to obtain a specificity of 90% and a lower 95% confidence interval (CI) of 80%.[10]

### Data sources and covariates

EMRALD is an advantageous data source for validating infection codes because it consists of all clinically relevant information from EMRs that can be linked to physician billing records within administrative databases. It has been used to validate other diseases.[11] EMRALD contains data for >400,000 patients who receive their primary care from a convenience sample of >350 family physicians distributed throughout Ontario who use the PS (Practice Solutions) Suite EMR. EMRALD contains clinical information such as a cumulative patient profile, progress notes, laboratory results, and prescriptions. Physicians participating in EMRALD are required to have had their EMR for ≥2 years to ensure it is adequately populated.

The Registered Persons Database contains basic demographic information on all individuals covered by provincial health insurance in Ontario (virtually the entire population) and was used to identify patient age, sex, and place of residence at the time of the physician office visit (index date). The child’s postal code was linked to Canadian census data to determine rural residence (communities with <10,000 residents), and quintile of neighbourhood material deprivation from the Ontario Marginalization Index, with 1 being the least deprived and 5 being the most deprived.[12, 13] The Ontario Health Insurance Plan (OHIP) database contains information on all physician billing claims, including diagnosis codes. Only one billing claim with an associated diagnosis code is processed for each service provided to the patient in the primary care setting. The diagnosis codes in OHIP are limited to 3 digits and is a truncated version of the International Classification of Diseases (ICD) 8 and 9.[14] The ICES Physician Database contains information on all physicians practicing in Ontario, and was used to obtain physician characteristics and specialization at the index date.

### Abstraction of EMR chart data

An abstraction manual and structured data collection form were created to identify and collect information about the infections by anatomic region and specific infectious syndromes. We selected a group of clinical syndromes that accounted for the majority of physician office visits for infections (Table 1). These infections were chosen a priori based on the knowledge gained from a systematic review and meta-analysis of common infections in children and the association with the development of childhood acute lymphoblastic leukemia.[15] We thought these infections would account for the majority of infection-related physician visits. We hierarchically defined each visit to assess whether the visit was for an infection, the corresponding anatomical region, and the specific infectious syndrome. Anatomic regions were respiratory, skin and soft tissue, gastrointestinal, urinary tract and otitis externa infections. The physician’s diagnosis must have reported one of the syndromes listed in Table 1 to be categorized as an infection. A diagnosis was not inferred if none was explicitly stated. The abstractor was blinded to the submitted diagnostic billing codes. We also abstracted any complex chronic conditions that impact health services utilization,[16] and other chronic conditions from the cumulative patient profile. Since the abstractor did not have clinical experience, and only one abstractor was used, we piloted the abstraction manual prior to full abstraction to clarify ambiguous situations, such as consultations with multiple diagnoses or complaints, and to measure the validity of the abstractor to correctly abstract the diagnoses from the medical charts. Diagnoses were abstracted verbatim from the medical charts to minimize subjective classifications. The results from the pilot were reviewed by co-authors with clinical experience to verify their validity. If multiple diagnoses were made, both were kept and compared to the corresponding billing code.

**Table 1:** The infections of interest from the electronic medical records and the corresponding Ontario Health Insurance Plan (OHIP) physician billing claim diagnosis codes.

Infections	OHIP diagnosis code
Respiratory infections	
• Upper respiratory infections or common cold	460
• Otitis media	381, 382
• Conjunctivitis	372
• Streptococcal sore throat	034
• Acute sinusitis	461
• Acute tonsillitis	463
• Acute laryngitis or croup	464
• Pertussis or whooping cough	033
• Infectious mononucleosis	075
Lower respiratory infections	486, 487, 466
• Pneumonia	486
• Influenza	487
• Acute bronchitis	466
Skin and soft tissue infections	
• Warts	078
• Impetigo	684
• Chalazion or sty	373
• Cellulitis	682
• Chicken pox or varicella	052
• Dental carries or dental abscess	521, 525
• Boils	680
• Herpes simplex	054
• Ringworm	110
• Candidiasis or thrush	112
Gastroenteritis or viral diarrhea	009
• Pinworm	127
Urinary tract infections	590, 595, 599
Otitis externa infection	380

### Analysis

Duplicate abstraction of a random sample of 200 patient visits was performed to assess intra-rater reliability. We calculated Cohen’s kappa, which measures the reliability of a single data collector who is presented with the same scenario interpreting the data and recording the same value.[17] We compared the demographic characteristics of the included and excluded patients using standardized differences and χ^2^ test for categorical variables, and one-way ANOVA test for mean age.[18] A standardized difference >0.10 indicates a potential imbalance in the prevalence of a variable between included and excluded patients. Diagnoses of infections in EMRALD were used as the reference standard and linked to the OHIP database. We calculated sensitivity, specificity, positive predictive value (PPV), and negative predictive value (NPV) for OHIP infection diagnosis codes occurring on the same day as the patient’s physician office visit. These measures are recommended for studies describing the diagnostic performance of administrative data for identifying diseases.[19] A binomial distribution was used for the performance measures to calculate 95% CI. We performed three sensitivity analyses to assess the performance measures based on: (1) if only one diagnosis was made, or a patient visited the physician for only one health complaint; (2) if multiple diagnoses were made at the time of the visit or a patient visited the physician for multiple complaints; and (3) patient characteristics stratified by age group, sex, rural versus urban residence, and presence of asthma and complex chronic conditions. All datasets were linked using unique, encoded identifiers, and were analyzed at ICES.

## Results

We identified 48,744 eligible patients of 251 physicians practising in 39 different clinics in EMRALD, and successfully abstracted data from 2,438 randomly sampled patients. After linkage to the administrative databases and applying the exclusions, 2,139 patients remained for analysis. We excluded 35 patients due to data quality concerns, such as being ineligible for OHIP at index date, and 264 patients due to the visit date on the EMR and the billing date in OHIP not aligning. Intra-rater reliability was almost perfect [k = 0.97 (95% CI 0.94–1.00)].

Characteristics of the patients and physicians in study cohort are summarized in Table 2. We observed a difference in rural residence, and in age groups 0 to <2 and 2 to 5 years between included and excluded patients (S1 Table). There were 2,185 unique OHIP billing claims in our cohort, and of those 1,669 (76.4%) EMR visit notes contained 1 diagnosis and 490 (22.4%) EMR visit notes contained multiple diagnoses. We found 33.3% of the visits in the EMR were for an infection. In mutually inclusive categories, respiratory infections accounted for 22.5% of all visits, skin and soft tissue infections for 8.3%, gastrointestinal infections for 2.0%, urinary tract infections for 1.3%, and otitis externa infections for 0.9%.

**Table 2:** Patient and physician characteristics of study cohort.

Characteristic	EMRALD patients, n (%)
Number of patients	2139
Female	1039 (48.6)
Age, average (SD)	6.7 (5.4)
0 to < 2	530 (24.8)
2 to 5	509 (23.8)
6 to 9	384 (18.0)
10 to 14	488 (22.8)
15 to 18	228 (10.7)
Rural residence	410 (19.2)
Material deprivation	
1 least	613 (28.7)
2	453 (21.2)
3	408 (19.1)
4	366 (17.2)
5 most	294 (13.8)
Chronic conditions or illnesses*	
Complex chronic conditions	77 (3.6)
Allergies	27 (1.3)
Asthma or reactive airways	203 (9.5)
Behavioral and emotional disorders with onset usually occurring in childhood and adolescence	144 (6.7)
Mood disorders	21 (1.0)
Pervasive and specific developmental disorders	48 (2.2)
**Physician Characteristics**	
Number of physicians	259
Female	145 (56.0)
Age, average (SD)	44.0 (10.7)
<35 years	71 (26.7)
35 to 44 years	85 (32.0)
45 to 54 years	58 (21.8)
55 to 75 years	52 (19.6)
Rural practice	26 (10.0)
Family physician or general practitioner	255 (98.5)
Canadian medical graduate	230 (88.8)
International medical graduate	29 (11.2)
Years of practice, average (IQR)	17.0 (7 to 26)

*Chronic conditions were identified through the electronic medical record’s cumulative patient profile; behavioural and emotional disorders, mood disorders and pervasive disorders were also identified through the cumulative patient profile as well as the diagnosis on the progress notes and were categorized based on International Classification of Disease-10 diseases categories. Material deprivation had 5 missing patients. SD represents standard deviation.

When we combined all infection categories, sensitivity was 0.74 (95% CI 0.70–0.77), specificity was 0.95 (95% CI 0.93–0.96), PPV was 0.87 (95% CI 0.84–0.90), and NPV was 0.88 (95% CI 0.86–0.89) (Table 3). Respiratory infections performed similarly with a sensitivity of 0.77 (95% CI 0.73–0.81), specificity of 0.96 (95% CI 0.95–0.97), PPV of 0.85 (95% CI 0.81–0.88), and NPV of 0.94 (95% CI 0.92–0.95). However, lower sensitivity was observed for skin and soft tissue, gastrointestinal, urinary tract, and otitis externa infections (0.42–0.53, Table 3). Specific infectious syndromes had sensitivity ranging from 0.32 to 1.00, PPV ranging from 0.50 to 1.00, specificity ranging from 0.96 to 1.00, and NPV ranging from 0.94 to 1.00 (Table 4). The sensitivity analyses suggested that almost all categories of infectious syndromes performed better if only one diagnosis was made or patients visited the physician for only one issue. Additional sensitivity analyses stratified by age group, sex, rural versus urban residence, asthma, and complex chronic conditions had similar performance to our primary analysis (S2 Table).

**Table 3:** Performance measures of the Ontario Health Insurance Plan physician billing claims for identifying infectious syndromes compared to electronic medical records.

Classification of infection	% infection in EMR	% infection in AD	Sensitivity [95% CI]	Specificity [95% CI]	PPV [95% CI]	NPV [95% CI]
**Performance of the different infections based on anatomic region, n = 2185**						
Any infection	33.3	28.1	74 (70–77)	95 (93–96)	87 (84–90)	88 (86–89)
Respiratory infection	22.5	20.5	77 (73–81)	96 (95–97)	85 (81–88)	94 (92–95)
Skin and soft tissue infection	8.3	4.8	49 (41–56)	99 (99–100)	86 (77–92)	96 (95–96)
Gastrointestinal infection	2.0	1.3	53 (38–69)	100 (99–100)	82 (63–94)	99 (99–99)
Urinary tract infections	1.3	1.0	50 (31–69)	100 (99–100)	64 (41–83)	99 (99–100)
Otitis externa infection	0.9	0.5	42 (20–67)	100 (100–100)	67 (35–90)	99 (99–100)
**Performance of different infections based on anatomic regions—Only 1 diagnosis was made at the visit, n = 1669**						
Any infection	30.4	27.4	79 (76–83)	95 (94–96)	88 (84–91)	91 (90–93)
Respiratory infection	20.3	20.1	84 (80–88)	96 (95–97)	85 (81–89)	96 (95–97)
Skin and soft tissue infection	7.3	5.0	57 (47–66)	99 (98–99)	82 (72–90)	97 (96–97)
Gastrointestinal infection	1.7	1.2	55 (36–74)	100 (99–100)	80 (56–94)	99 (99–100)
Urinary tract infections	0.7	0.8	73 (39–94)	100 (99–100)	62 (32–86)	100 (99–100)
Otitis externa infection	0.6	0.4	50 (19–81)	100 (100–100)	83 (36–100)	100 (99–100)
**Performance of different infections based on anatomic regions—Multiple diagnoses was made at the visit, n = 490**						
Any infection	44.9	30.8	61 (54–67)	94 (90–96)	89 (83–93)	75 (70–79)
Respiratory infection	31.0	22.0	62 (54–70)	96 (93–98)	87 (79–93)	85 (81–88)
Skin and soft tissue infection	12.2	4.1	33 (22–47)	100 (99–100)	100 (83–100)	91 (89–94)
Gastrointestinal infection	2.7	1.6	54 (25–81)	100 (99–100)	88 (47–100)	99 (97–100)
Urinary tract infections	3.5	1.8	35 (14–62)	99 (98–100)	67 (30–93)	98 (96–99)
Otitis externa infection	1.8	1.2	33 (7–70)	99 (98–100)	50 (12–88)	99 (97–100)

EMR = electronic medical records, AD = administrative data, PPV = positive predictive value, NPV = negative predictive value.

**Table 4:** Performance measures of the Ontario Health Insurance Plan physician billing claims for identifying specific infectious syndromes compared to electronic medical records.

Classification of infectious syndrome	% infection in EMR	% infection in AD	Sensitivity [95% CI]	Specificity [95% CI]	PPV [95% CI]	NPV [95% CI]
Upper respiratory infection + conjunctivitis + otitis media	18.9	17.8	75 (71–80)	96 (94–96)	80 (75–84)	94 (93–95)
Upper respiratory infection (Pharyngitis, sinusitis, tonsillitis, laryngitis, or streptococcal sore throat)	13.6	11.9	69 (63–74)	97 (96–98)	79 (73–84)	95 (94–96)
Otitis media	4.7	4.4	72 (62–80)	99 (98–99)	77 (67–85)	99 (98–99)
Conjunctivitis	1.4	1.6	77 (58–90)	99 (99–100)	68 (49–83)	100 (99–100)
Strep throat	2.2	1.0	32 (19–47)	100 (99–100)	71 (48–89)	99 (98–99)
Bronchitis	0.6	0.7	64 (35–87)	100 (99–100)	56 (30–80)	100 (99–100)
Croup or laryngitis	0.8	0.4	41(18–67)	100 (100–100)	88 (47–100)	100 (99–100)
Tonsillitis	0.5	0.6	70 (35–93)	100 (99–100)	50 (23–77)	100 (100–100)
Sinusitis	0.5	0.5	73 (39–94)	100 (100–100)	67 (35–90)	100 (100–100)
Infectious mononucleosis	0.5	<0.03	40 (12–74)	100 (100–100)	100 (40–100)	100 (99–100)
Lower respiratory infection (unspecified lower respiratory infection, pneumonia, influenza, or acute bronchitis)	3.1	2.4	62 (49–73)	100 (99–100)	81 (67–90)	99 (98–99)
Pneumonia	2.0	1.3	60 (44–75)	100 (100–100)	90 (73–98)	99 (99–100)
Warts	2.7	2.1	69 (55–80)	100 (99–100)	87 (74–95)	99 (99–100)
Impetigo	1.0	0.7	59 (36–79)	100 (100–100)	87 (60–98)	100 (99–100)
Chalezon or stye	0.6	0.04	54 (25–81)	100 (100–100)	88 (47–100)	100 (99–100)
Cellulitis	0.5	0.5	55 (23–83)	100 (100–100)	60 (26–88)	100 (99–100)
Gastroenteritis, viral diarrhea, or viral gastritis	1.7	1.2	59 (42–75)	100 (99–100)	81 (62–94)	99 (99–100)
Urinary tract infections	1.3	1.0	50 (31–69)	100 (99–100)	64 (41–83)	99 (99–100)

Infectious syndromes with ≤10 events from the electronic medical record are not reported. EMR = electronic medical records, AD = administrative data, PPV = positive predictive value, NPV = negative predictive value. Cells suppressed because of small cell size (direct or by inference).

## Discussion

Overall, we found that using linked EMR data as the reference standard, administrative billing codes are valid to identify infections in a pediatric population. The approach of measuring infections using administrative data performed best when the patient visited the physician with only one health complaint or if only one diagnosis was made. Administrative data performed well in capturing any infection and respiratory infections, while skin and soft tissue, gastrointestinal, urinary tract, and other ear infections maintained high specificity, but had lower sensitivity. Performance characteristics were similar among children with chronic diseases and complex chronic conditions. These results suggest administrative data can accurately capture infections with minimal risk of including false positives.

Other validation studies of administrative data to measure infections have shown consistent findings with our study.[20–26] These studies assessed hospitalizations or emergency room visits for respiratory infections, respiratory syncytial virus, rotavirus, pneumonia, skin infection, *Clostridium difficile* infection, and urinary tract infections. They found poor-to-high sensitivity (45% to 99%), moderate-to-high specificity (69% to 100%), poor-to-high PPV (55% to 100%), and had to trade-off higher sensitivity for lower specificity or vice versa by expanding the number of ICD diagnosis codes, the number of data fields, or the diagnosis types. Our estimates for any infection, respiratory infection, and specific infectious syndromes such as otitis media and conjunctivitis performed well compared to these studies.

We found infections accounted for 33.3% of all visits to a physician, respiratory infections accounted for 67.6% of those infections. Followed by skin and soft tissue infections represented a quarter of the visits for an infection, gastrointestinal infections represented 6.0%, urinary tract infections represented 3.9%, and otitis externa represented 2.7%. Infections continue to represent one of the most frequent reasons to seek healthcare in children and adolescents aged <18 years.[1–4, 27]

This study utilizes a population-based primary care cohort and is the largest to date that gives evidence on the diagnostic performance of administrative data in identifying infections in children within a primary care setting. The contributions of the study are important for a field with limited evidence and demonstrates the validity of administrative data in identifying infections in children for clinicians, researchers, and decision makers. This will allow for future studies in this area to examine larger populations and changes over time. However, our study had several limitations. First, only one abstractor without clinical experience was used and this could have implications on the validity of the study. However, our pilot demonstrated that one abstractor was able to abstract the diagnoses from the medical charts accurately and reliably. Second, our reference standard relied on the physician’s clinical judgement and completeness of documentation. Third, we did not use laboratory confirmation to identify specific infectious agents. It is not known how well the syndromic data correlate with microbiological test results. However, a study in an emergency department setting demonstrated that respiratory syndrome diagnosis counts were associated with positive viral tests for infectious respiratory agents and showed that the rate of respiratory syncytial virus and influenza virus was positively associated with respiratory syndrome counts (rate ratio = 1.51, 95% CI 1.10–2.07).[28] Another limitation is that there were differences between those included and excluded in this study and this may have implications for generalizability of the study.

The data available through EMRALD are from a voluntary sample of physicians in Ontario who all use one type of EMR system and practice under one of the primary care reform models; therefore the results of this study might not be generalizable to other physicians. EMRALD physicians were found to be younger, more likely to be female, to be a Canadian medical graduate and to participate in patient-enrolment models compared to the general physician population in Ontario.[29] However, this likely reflects the characteristics of physicians who have adopted EMR software and trends in the primary care workforce. Ontario has been undergoing a primary care reform for more than a decade where the new primary care models require ‘rostering’ of patients (patient-enrollment models) and the physician acts as the their most responsible physician.[30] Although patients rostered in EMRALD are more likely to live in rural areas and be of higher socioeconomic status; the age, sex, presence of chronic conditions and measures of comorbidity are similar to rostered patients in Ontario.[31] The differences in physician characteristics between EMRALD and Ontario are unlikely to bias the internal validity of the study. While our findings provide insight into the validity of administrative data to identify infectious syndromes in Ontario, they may not be generalizable to Ontario specialists or family physicians not participating in EMRALD, or to other jurisdictions where physician billing practices or disease classification systems may differ. An important limitation is that this study was conducted in Canada and our results may not be generalizable to other countries. However, they are more likely to be applicable to countries with similar healthcare systems and more specifically, studies conducted in the United States and New Zealand showed consistent findings in the performance of the administrative data to identify infections.[21–26]

Our study demonstrates the diagnostic performance of a viable method to identify syndromic conditions for the use of syndrome-based burden of disease estimates using healthcare administrative data. Future priorities could include the development of a surveillance system using EMR data as demonstrated in other studies.[32] Other priorities could include investigations of factors, needs and healthcare barriers that contribute to inequalities in healthcare in vulnerable populations. For example, infectious diseases in children contribute substantially to healthcare utilization in primary care physician offices and at emergency departments. The associated annual cost for emergency department visits for infections was almost $10 billion in the United States in 2011.[33] However, the proportion of healthcare utilization for infections was disproportionally higher in children of lower socioeconomic status in the emergency department, but was lower in primary care offices.[27, 33] Studies that address the potential needs, factors, and barriers to healthcare utilization are required to inform decision-makers of the most cost-effective, impactful population-based preventive interventions, and for resource planning.

## Supporting information

S1 File STROBE Statement—Checklist of items that should be included in reports of observational studies. *Give information separately for cases and controls in case-control studies and, if applicable, for exposed and unexposed groups in cohort and cross-sectional studies.
(DOC)

S1 Table Patient characteristics of those excluded from the analysis due to misalignment of the visit date on the electronic medical record and the billing date in Ontario Health Insurance Plan. There are 9 missing individuals in the residential instability, material deprivation, dependency, and ethnic concentration variables. Standardized difference >0.10 indicates an imbalance in the prevalence of the covariate between the included and excluded patients. A p-value >0.05 in the χ^2^ test indicates a difference between included and excluded patients. One-way ANOVA test was used for mean age comparison. Some cells (≤5) suppressed because of small cell size (direct or by inference), which cannot be reported as per privacy regulations.
(DOCX)

S2 Table Performance measures of the Ontario Health Insurance Plan physician billing claims for identifying infectious syndromes compared to electronic medical records, by age group, sex, rural and urban residence, presence of asthma or reactive airways, and presence of chronic complex conditions. *Cells suppressed because of small cell size (direct or by inference), which cannot be reported as per privacy regulations, and performance characteristics have deliberately not been reported due to the potential to back-calculate the small cell sizes. Cells with ≤5 persons have been suppressed. EMR = electronic medical records, AD = administrative data, PPV = positive predictive value, NPV = negative predictive value.
(DOCX)
