# Supplementary material for: Use of physician billing claims to identify infections in children
Source: PLoS One. 2018 Nov 12;13(11):e0207468. doi: 10.1371/journal.pone.0207468 (PMC6231686; doi:10.1371/journal.pone.0207468)
Supplement: S1 Table — There are 9 missing individuals in the residential instability, material deprivation, dependency, and ethnic concentration variables. Standardized difference >0.10 indicates an imbalance in the prevalence of the covariate between the included and excluded patients. A p-value >0.05 in the χ2 test indicates a difference between included and excluded patients. One-way ANOVA test was used for mean age comparison. Some cells (≤5) suppressed because of small cell size (direct or by inference), which cannot be reported as per privacy regulations. (DOCX) [file pone.0207468.s002.docx]

S1 Table. Patient characteristics of those excluded from the analysis due to misalignment of the visit date on the electronic medical record and the billing date in Ontario Health Insurance Plan.

| **Characteristic** | **EMRALD patients, n (%)** | **Standardized difference or p-value for comparison to cohort of included patients** |
| --- | --- | --- |
| Number of patients | 264 |  |
| Female | 118 (44.7) | p=0.23 |
| Age, average (SD) | 7.1 (5.3) | p=0.08 |
| 0 to < 2 | 45 (17.0) | 0.19 |
| 2 to 5 | 75 (28.4) | 0.11 |
| 6 to 9 | 48 (18.2) | 0.01 |
| 10 to 14 | 65 (24.6) | 0.04 |
| 15 to 18 | 31 (11.7) | 0.03 |
| Rural residence | 73 (27.7) | p<0.01 |
| Residential instability |  |  |
| 1 least | 48 (18.2) | 0.06 |
| 2 | 58 (22.0) | 0 |
| 3 | 52 (19.7) | 0.04 |
| 4 | 53 (20.1) | 0.01 |
| 5 most | 44 (16.7) | 0.07 |
| Material deprivation |  |  |
| 1 least | 69 (26.1) | 0.06 |
| 2 | 52 (19.7) | 0.04 |
| 3 | 44 (16.7) | 0.06 |
| 4 | 53 (20.1) | 0.08 |
| 5 most | 37 (14.0) | 0.01 |
| Dependency |  |  |
| 1 least | 79 (29.9) | 0.03 |
| 2 | 60 (22.7) | 0.07 |
| 3 | 42 (15.9) | 0.05 |
| 4 | 37 (14.0) | 0.02 |
| 5 most | 37 (14.0) | 0.03 |
| Ethnic concentration |  |  |
| 1 least | 51 (19.3) | 0.08 |
| 2 | 50 (18.9) | 0.04 |
| 3 | 54 (20.5) | 0.03 |
| 4 | 60 (22.7) | 0.08 |
| 5 most | 40 (15.2) | 0.01 |
| Chronic conditions or illnesses | |  |
| Complex Chronic Conditions | 7 (2.7) | p=0.43 |
| Allergies | ≤5 | p=0.73 |
| Asthma or reactive airways | 14 (7.0) | p=0.16 |
| Behavioral and emotional disorders with onset usually occurring in childhood and adolescence | 10 (5.0) | p=0.52 |
| Mood disorders | ≤5 | p=0.72 |
| Pervasive and specific developmental disorders | ≤5 | p=0.72 |

There are 9 missing individuals in the residential instability, material deprivation, dependency, and ethnic concentration variables. Standardized difference >0.10 indicates an imbalance in the prevalence of the covariate between the included and excluded patients. A p-value >0.05 in the χ^2^ test indicates a difference between included and excluded patients. One-way ANOVA test was used for mean age comparison. Some cells (≤5) suppressed because of small cell size (direct or by inference), which cannot be reported as per privacy regulations.
