# Supplementary material for: Use of physician billing claims to identify infections in children
Source: PLoS One. 2018 Nov 12;13(11):e0207468. doi: 10.1371/journal.pone.0207468 (PMC6231686; doi:10.1371/journal.pone.0207468)
Supplement: S2 Table — *Cells suppressed because of small cell size (direct or by inference), which cannot be reported as per privacy regulations, and performance characteristics have deliberately not been reported due to the potential to back-calculate the small cell sizes. Cells with ≤5 persons have been suppressed. EMR = electronic medical records, AD = administrative data, PPV = positive predictive value, NPV = negative predictive value. (DOCX) [file pone.0207468.s003.docx]

S2 Table. Performance measures of the Ontario Health Insurance Plan physician billing claims for identifying infectious syndromes compared to electronic medical records, by age group, sex, rural and urban residence, presence of asthma or reactive airways, and presence of chronic complex conditions.

|  | **Classification of infection** | **% infection in EMR** | **% infection in AD** | **Sensitivity [95% CI]** | **Specificity [95% CI]** | **PPV  [95% CI]** | **NPV [95% CI]** |
| --- | --- | --- | --- | --- | --- | --- | --- |
| **Age 0-2, n=546** | Any infection | 22.5 | 20.1 | 76 (67-83) | 96 (94-98) | 85 (76-91) | 93 (90-95) |
|  | Respiratory infection | 19.0 | 17.0 | 78 (69-85) | 97 (95-99) | 87 (79-93) | 95 (92-97) |
|  | Skin and soft tissue infection | 2.0 | 1.1 | 27 (6-61) | 99 (98-100) | 50 (12-88) | 99 (97-99) |
|  | Gastrointestinal infection | 2.0 | 1.6 | 64 (31-89) | 100 (99-100) | 78 (40-97) | 99 (98-100) |
|  | Urinary tract infections | 0.0 | 0.0 |  |  |  |  |
|  | Otitis externa (ear) infection | 0.0 | ≤1.0* |  |  |  |  |
| **Age 2-5, n=519** | Any infection | 44.9 | 39.5 | 78 (72-83) | 92 (88-95) | 88 (83-92) | 83 (79-87) |
|  | Respiratory infection | 33.7 | 31.0 | 78 (71-84) | 93 (89-95) | 84 (78-90) | 89 (85-92) |
|  | Skin and soft tissue infection | 7.7 | 4.8 | 55 (38-71) | 99 (98-100) | 88 (69-97) | 96 (94-98) |
|  | Gastrointestinal infection | 2.7 | 1.9 | 57 (29-82) | 100 (99-100) | 80 (44-97) | 99 (97-100) |
|  | Urinary tract infections | 1.9 | 1.3 | 50 (19-81) | 100 (99-100) | 71 (29-96) | 99 (99-100) |
|  | Otitis externa (ear) infection | ≤1.0* | ≤1.0* |  |  |  |  |
| **Age 6-9, n=390** | Any infection | 41.3 | 36.4 | 78 (70-84) | 93 (88-96) | 88 (82-93) | 85 (80-90) |
|  | Respiratory infection | 24.1 | 23.3 | 79 (69-86) | 94 (91-97) | 81 (72-89) | 93 (90-96) |
|  | Skin and soft tissue infection | 13.1 | 9.5 | 67 (52-79) | 99 (97-100) | 92 (78-98) | 95 (92-97) |
|  | Gastrointestinal infection | 2.6 | ≤1.4* |  |  |  |  |
|  | Urinary tract infections | 2.8 | 2.1 | 55 (23-83) | 99 (98-100) | 75 (35-97) | 99 (97-100) |
|  | Otitis externa (ear) infection | 1.5 | ≤1.4* |  |  |  |  |
| **Age 10-14, n=497** | Any infection | 31.0 | 24.1 | 66 (58-74) | 95 (92-97) | 85 (77-91) | 86 (82-90) |
|  | Respiratory infection | 17.9 | 16.5 | 76 (66-85) | 97 (94-98) | 83 (73-90) | 95 (92-97) |
|  | Skin and soft tissue infection | 12.5 | 5.2 | 35 (24-49) | 99 (98-100) | 85 (65-96) | 92 (89-94) |
|  | Gastrointestinal infection | ≤1.0* | ≤1.0* |  |  |  |  |
|  | Urinary tract infections | ≤1.0* | ≤1.0* |  |  |  |  |
|  | Otitis externa (ear) infection | 1.6 | ≤1.0* |  |  |  |  |
| **Age 15+, n=233** | Any infection | 24.5 | 15.9 | 61 (48-74) | 99 (96-100) | 95 (82-99) | 89 (84-93) |
|  | Respiratory infection | 12.9 | 9.0 | 70 (51-85) | 100 (98-100) | 100 (84-100) | 96 (92-98) |
|  | Skin and soft tissue infection | 7.7 | 4.3 | 44 (22-69) | 99 (97-100) | 80 (44-97) | 96 (92-98) |
|  | Gastrointestinal infection | ≤2.15* | ≤2.15* |  |  |  |  |
|  | Urinary tract infections | ≤2.15* | ≤2.15* |  |  |  |  |
|  | Otitis externa (ear) infection | ≤2.15* | ≤2.15* |  |  |  |  |
| **Female, n=1066** | Any infection | 33.5 | 26.9 | 71 (66-76) | 95 (94-97) | 89 (84-92) | 87 (84-89) |
|  | Respiratory infection | 21.7 | 19.3 | 77 (71-82) | 97 (95-98) | 86 (81-91) | 94 (92-95) |
|  | Skin and soft tissue infection | 8.3 | 4.1 | 42 (31-53) | 99 (99-100) | 84 (70-93) | 95 (93-96) |
|  | Gastrointestinal infection | 2.7 | 1.4 | 48 (29-67) | 100 (99-100) | 93 (68-100) | 99 (98-99) |
|  | Urinary tract infections | 2.1 | 1.3 | 45 (24-68) | 100 (99-100) | 71 (42-92) | 99 (98-100) |
|  | Otitis externa (ear) infection | 1.0 | 0.8 | 45 (17-77) | 100 (99-100) | 63 (24-91) | 99 (99-100) |
| **Male, n=1119** | Any infection | 33.2 | 29.2 | 76 (71-80) | 94 (92-96) | 86 (82-90) | 89 (86-91) |
|  | Respiratory infection | 23.3 | 21.6 | 77 (72-82) | 95 (94-97) | 83 (78-88) | 93 (91-95) |
|  | Skin and soft tissue infection | 8.3 | 5.4 | 56 (45-66) | 99 (98-100) | 87 (75-94) | 96 (95-97) |
|  | Gastrointestinal infection | 1.3 | 1.2 | 64 (35-87) | 100 (99-100) | 69 (39-91) | 100 (99-100) |
|  | Urinary tract infections | ≤0.5* | 0.7 |  |  |  |  |
|  | Otitis externa (ear) infection | 0.7 | ≤0.5* |  |  |  |  |
| **Rural, n=416** | Any infection | 41.1 | 34.4 | 75 (68-81) | 94 (90-97) | 90 (83-94) | 84 (79-88) |
|  | Respiratory infection | 27.4 | 24.0 | 75 (66-82) | 95 (92-97) | 85 (76-91) | 91 (87-94) |
|  | Skin and soft tissue infection | 11.5 | 7.9 | 60 (45-74) | 99 (97-100) | 88 (72-97) | 95 (92-97) |
|  | Gastrointestinal infection | 2.4 | 1.4 | 60 (28-88) | 100 (99-100) | 100 (54-100) | 99 (98-100) |
|  | Urinary tract infections | ≤1.2* | ≤1.2* |  |  |  |  |
|  | Otitis externa (ear) infection | ≤1.2* | ≤1.2* |  |  |  |  |
| **Urban, n=1767** | Any infection | 31.5 | 26.7 | 73 (69-77) | 95 (93-96) | 87 (83-90) | 89 (87-90) |
|  | Respiratory infection | 21.4 | 19.7 | 78 (74-82) | 96 (95-97) | 85 (81-88) | 94 (93-95) |
|  | Skin and soft tissue infection | 7.6 | 4.0 | 45 (36-54) | 99 (99-100) | 85 (74-92) | 96 (95-97) |
|  | Gastrointestinal infection | 1.9 | 1.2 | 52 (34-69) | 100 (99-100) | 77 (55-92) | 99 (99-99) |
|  | Urinary tract infections | 1.4 | 1.1 | 50 (29-71) | 100 (99-100) | 60 (36-81) | 99 (99-100) |
|  | Otitis externa (ear) infection | 1.0 | 0.6 | 41 (18-67) | 100 (100-100) | 70 (35-93) | 99 (99-100) |
| **Asthma or reactive airways, n=210** | Any infection | 34.2 | 31.2 | 74 (62-84) | 91 (86-96) | 81 (69-90) | 88 (81-93) |
|  | Respiratory infection | 22.4 | 21.9 | 74 (60-86) | 93 (88-97) | 76 (61-87) | 93 (88-96) |
|  | Skin and soft tissue infection | 9.0 | 4.8 | 47 (24-71) | 99 (97-100) | 90 (56-100) | 95 (91-98) |
|  | Gastrointestinal infection | ≤2.4* | ≤2.4* |  |  |  |  |
|  | Urinary tract infections | ≤2.4* | ≤2.4* |  |  |  |  |
|  | Otitis externa (ear) infection | ≤2.4* | ≤2.4* |  |  |  |  |
| **No asthma or reactive airways, n=1975** | Any infection | 33.4 | 27.9 | 74 (70-77) | 95 (94-96) | 88 (85-91) | 88 (86-89) |
|  | Respiratory infection | 22.5 | 20.4 | 78 (73-81) | 96 (95-97) | 86 (82-89) | 94 (92-95) |
|  | Skin and soft tissue infection | 8.3 | 4.8 | 49 (41-57) | 99 (99-100) | 85 (76-92) | 96 (95-96) |
|  | Gastrointestinal infection | 2.1 | 1.3 | 54 (37-69) | 100 (99-100) | 85 (65-96) | 99 (98-99) |
|  | Urinary tract infections | 1.3 | 1.0 | 50 (30-70) | 100 (99-100) | 65 (41-85) | 99 (99-100) |
|  | Otitis externa (ear) infection | 0.8 | 0.5 | 38 (15-65) | 100 (100-100) | 67 (30-93) | 99 (99-100) |
| **Complex Chronic Conditions, n=78** | Any infection | 24.4 | 21.8 | 79 (54-94) | 97 (88-100) | 88 (64-99) | 93 (84-98) |
|  | Respiratory infection | 20.5 | 17.9 | 75 (48-93) | 97 (89-100) | 86 (57-98) | 94 (85-98) |
|  | Skin and soft tissue infection | ≤6.4* | ≤6.4* |  |  |  |  |
|  | Gastrointestinal infection | ≤6.4* | ≤6.4* |  |  |  |  |
|  | Urinary tract infections | ≤6.4* | ≤6.4* |  |  |  |  |
|  | Otitis externa (ear) infection | 0.0 | 0.0 |  |  |  |  |

*Cells suppressed because of small cell size (direct or by inference), which cannot be reported as per privacy regulations, and performance characteristics have deliberately not been reported due to the potential to back-calculate the small cell sizes. Cells with ≤5 persons have been suppressed. EMR=electronic medical records, AD=administrative data, PPV=positive predictive value, NPV=negative predictive value.
